# An epidemiological study of prevalence and comorbidity of obsessive compulsive disorder symptoms (SOCD) and stress in Pakistani Adults

**DOI:** 10.12669/pjms.334.13045

**Published:** 2017

**Authors:** Farzana Ashraf, Sadia Malik, Amna Arif

**Affiliations:** 1 Dr. Farzana Ashraf, PhD. Department of Humanities, COMSATS Institute of Information Technology, Lahore, Pakistan; 2 Dr. Sadia Malik, PhD. Department of Psychology, University of Sergodha, Sergodha, Pakistan; 3 Ms. Amna Arif, M.Phil. Department of Special Education, University of Management & Technology, Lahore, Pakistan

**Keywords:** Comorbidity, Employment Status, Marital Status, Prevalence, SOCD, Stress

## Abstract

**Objective:**

To investigate the prevalence and comorbidity of subclinical obsessive compulsive disorder (SOCD) symptoms and stress across gender, marital and employment statuses.

**Methods:**

A cross-sectional research was conducted from December, 2016 to March 2017 at two universities of cosmopolitan city Lahore. Two self-report scales measuring SOCD symptoms and stress were used to collect data from 377 adults selected through simple random sampling technique, proportionately distributed across gender, marital and employment status.

**Results:**

From the total sample, 52% reported low level of stress and 48% faced high level of stress. Significant differences in prevalence were observed across marital and employment statuses whereas for men and women, it was observed same (24%). Comorbidity of high level of SOCD symptoms and high level of stress was seen 34%.

**Conclusion:**

Significant prevalence and comorbidity exists between SOCD symptoms and stress and more studies addressing diverse population are needed.

## INTRODUCTION

Obsessive compulsive disorder (OCD) is a chronic psychiatric disorder classified by recurrent intrusive thoughts and/or repetitive compulsive behaviors. Subclinical OCD (SOCD) is characterized by obsessive or compulsive symptoms or both. These symptoms do not meet the required duration, intensity/frequency and severity of clinical impairment. For clinical samples, the DSM-5^1^ documented a general prevalence of OCD from 1.1% to 1.8%. In case of subclinical samples, a general prevalence of SOCD has been observed as 12.3%.^2^

Overall, very few empirical investigations concerning SOCD had been carried out. Past research illustrated the frequent prevalence of SOCD symptoms than the clinical nature of OCD in the general population.^3^ DSM-5 documented that gender might play a role in the onset and nature of OCD symptoms in adults as women show a higher prevalence of OCD symptoms as compared to their counterparts. Moreover, women are more likely to demonstrate acute onset of SOCD, as in a Turkish normative sample of adults, the ratio of women to men was observed 3.3% to 2.5%.^4^ Contrary to that, in a German study, the ratio of SOCD in women was 1% as compared to 2% in men.^5^

Stress is common and experienced as a state of strain characterized by tension and emotional experiences resulting from adverse circumstances. Previously, stress has been widely examined across general, subclinical and psychiatric or clinical populations. According to a cross sectional survey, 51% women compared to 43% of men reported high level of stress.^6^ In a normative sample, women demonstrated high prevalence of stress symptoms than their counterparts.^7^ Stress varies across marital statuses as a recent study claimed that unmarried people face high level of stress than married and this could be due to the high level of cortisol which is seen low in married participants.^8^ Along with gender and marital status, work status also reported in association with stress as employed adults exhibited low level of stress.^9^

Comorbidity of clinical OCD with several other disorders e.g., anxiety (76%) and depressive/bipolar disorder (63%) has been documented.^1^ A large number of clinical case reports identify the unique direction of coexistence of obsessive compulsive disorder (OCD) symptoms with stress. Therefore, it’s well justified to assume comorbidity of SOCD with stress as SOCD is more common than OCD. Contrary to case studies, empirical and epidemiological studies reported mixed finding.^10^ Women with high SOCD symptoms report a heightened self-reported stress. Whereas for men, mixed findings were documented.^11^ In the local perspective, somehow SOCD has been explored in academically, socially and economically deprived group of participants.^12^ No empirical data have been documented in educated normative samples on the prevalence of SOCD in general and its comorbidity with stress in specific. It could be due to the fact that in Pakistan, mental/psychiatric health problems are not perceived as problematic. It also clarifies the understanding that the prevalence of SOCD in general or subclinical population remains unrecognized and underestimated.

Although, limited research has examined this direction in the general population. However, some reported significant positive associations between SOCD and stress.^13^ In addition to gender, high prevalence of SOCD symptoms has been noticed in relation to several other personal factors such as marital status and employed status.^14^

The current research was designed to achieve two objectives;

- To examine the prevalence of SOCD symptoms and stress across genders, marital and work statuses.
- To identify the comorbidity of SOCD symptoms with stress across genders, marital and work statuses.

## METHODS

It was a cross-sectional research conducted at two universities of Lahore (COMSATS Institute of Information Technology and University of Management and Technology). The present research was conducted over a period of 4 months which was initiated in December 2016. The participants were selected through convenience sampling and recruited after they provided their informed consent.

### Study Sample

The sample comprised of 377 educated adults (men=181, women=196) ages between 18 to 46 years (*M*=27.27, *SD*=±6.08). Participants’ qualification ranges between intermediate to PhD (*M*=16.12, *SD*=±1.97). The response rate was 94% because the participants were well qualified and realized the importance of participating in a research. The sample size was determined by performing Raosoft website’s calculations, with 5% margin of error, 50% response distribution and 95% confidence interval. The sample of 377 is further classified into employed= 198 non-employed=179, and marital status (married=196, unmarried=181). Non-employed participants comprised of students whereas employed sample consisted of teachers, assistant teachers, campus counselors, administrators, coordinators and helping staff.

### Tools

The participants were administered Padua Inventory of Obsessive Compulsion Disorder Symptoms^15^ and Perceived Stress Scale.^16^ The internal consistency for both scale was calculated through Cronbach’s Alpha Coefficients and found 0.87 and 0.75 respectively. Prevalence of high/low levels of SOCD symptoms and stress (high/low) across study categorical variables (e.g., gender, marital status and employment status) were assessed by using Chi square test of association. For the present study, p-value of </=.05 was determined to report statistical significance of findings (Table-I).

**Table-I:** Prevalence of SOCD Symptoms and Stress across Gender, Marital and Work Statuses (N=377).

*Variables*		*Gender*			*Marital Status*			*Work Status*		

	*Overall Sample*	*Men f (%)*	*Women f (%)*	*χ*	*Married f (%)*	*Unmarried f (%)*	*χ*	*Employed f (%)*	*Non-employed f (%)*	*χ*
SOCD				1.80			4.70*			25.51***
Low	201(53%)	90(24%)	111(29%)		115(30%)	81(21%)		130(34%)	71(19%)	
High	176(47%)	91(24%)	85(23%)		86(22%)	95(25%)		68(18%)	108 (29%)	
1- Contamination & Washing				3.67*			5.02*			6.58*
Low Scores	212(56%)	111(29%)	101(27%)		121(32%)	91(24%)		99(26%)	113(30%)	
High Scores	165(44%)	70(19%)	95(25%)		74(18%)	91(24%)		99(26%)	66(18%)	
2- Dressing				.51			7.74**			.49
Low Scores	174(46%)	87(23%)	94(25%)		77(20%)	119(31%)		88(23%)	85(23%)	
High Scores	203(54%)	87(23%)	109(29%)		97(26%)	84(23%)		111(29%)	93(25%)	
3- Checking & Compulsions				.88			42.08***			70.88***
Low Scores	107(28%)	52(13%)	55(14%)		84(23%)	112(30%)		93(25%)	14(4%)	
High Scores	270(72%)	129(34%)	141(39%)		23(6%)	158(41%)		105(28%)	165(43%)	
4- Obsessional thoughts of Harm				4.37*			10.84**			9.41**
Low Scores	206(55%)	109(29%)	97(26%)		123(33%)	73(20%)		123(33%)	83(22%)	
High Scores	171(45%)	72(19%)	99(26%)		83(22%)	98(25%)		75(20%)	96(25%)	
5- Obsessional Impulses				40.97***			.40			7.08**
Low Scores	204(54%)	67(17%)	137(37%)		103(28%)	93(24%)		120(32%)	84(22%)	
High Scores	173(46%)	114(30%)	59(16%)		101(27%)	80(21%)		78(21%)	95(25%)	
Stress							2.81			49.55***
Low Scores	194(51%)	90(24%)	104(28%)	.42	109(29%)	85(23%)		136(36%)	58(15%)	
High Scores	183(49%)	91(24%)	92(24%)		87(23%)	96(25%)		62(16%)	121(33%)	

* p<.05,

** p<.01,

*** p<.001

### Ethical considerations

This study was approved by Ethical Review Board of Department of Humanities, COMSATS Institute of Information Technology, Lahore. Later, participants were informed about the purpose of study and importance of their participation. They were informed that their participation is voluntary and they can withdraw from it at any point during the study.

## RESULTS

In sample of 377 adults, high to low level of stress ratio was 52% to 48%. For SOCD symptoms, it was found 53% to 47%. The participants with high or low stress and SOCD symptoms were classified by running Receiver Operating Characteristic curve analysis.

### Comparison of men with women

The cross tab analysis didn’t reveal significant differences in prevalence of stress and SOCD symptoms in men and women. Partially, SOCD symptoms varied across genders such as contamination and washing was significantly high in women (25%) as compared to men (19%). Prevalence of obsessional thoughts of harm were more in women (26%) than in men (19%). Table-I. Contrary to that, obsessional impulses were noticed significantly high (χ =40.97, *p*<.001) in men (30%) than their counterparts (16%). No gender differences in prevalence of low or high symptoms of dressing and, checking and compulsion were seen.

### Comparison across Marital Status

The prevalence of stress was not found significantly different across marital status (married and unmarried). Whereas, high SOCD symptoms were more prevalent in unmarried participants (25%) as compare to married (22%). Interestingly, mix results were seen on measures of SOCD. High symptoms of contamination and washing, checking and compulsions and obsessional thoughts of harm were more prevalent for unmarried sample (24%, 41% and 25% respectively) as compared to married (18%, 6%, and 22% respectively). Other measures of SOCD e.g., symptoms of dressing and obsessional impulses were more prevalent in married adults (26% and 27% respectively) than unmarried (Table-I).

### Comparison across Employment Status

In case of employment status, non-employed participants reported significant (*p*<.001) high level of stress (33%) and SOCD symptoms (19%) than the comparison group (employed group; 16% and 18% respectively). On one hand, symptoms of contamination and washing, and dressing were high in employed participants (26%, 29% respectively) as compare to non-employed (18% and 25% respectively). On the other hand, checking and compulsion, obsessional thoughts of harm and obsessional impulses were observed high in non-employed individuals (43%, 25% and 25% respectively) than for employed (28%, 20% and 21% respectively).

Table-II shows significant comorbidity (χ=39.46, *p*=.000) of SOCD symptoms with stress as 31% of the study sample reported high level of stress and SOCD symptoms. In men, this comorbidity was shown 38%. For sample of women, it was comparatively low (24%). Among married participants, 31% reported coexistence of high level of stress and SOCD symptoms. Similar findings were observed for the sample of unmarried participants. In case of employed group, 20% exhibited high level of stress and SOCD symptoms. For non-employed group, analysis indicated it as 43%.

**Table-II:** Comorbidity between SOCD Symptoms and Stress.

*SOCD*	*Stress*					

*Overall Sample*	*Low Scores (f %)*	*High Scores (f %)*	*χ (f %)*	*Low Scores (f %)*	*High Scores*	*χ*
Low Scores (f %)	134(35%)	67(18%)	39.46***	-	-	-
High Scores (f %)	60(16%)	116(31%)		-	-	-
	Men (n=181)			Women (n=196)		
Low Scores (f %)	68(38%)	22(12%)	47.78***	66(34%)	45(23%)	4.20*
High Scores (f %)	22(12%)	69(38%)		38(19%)	47(24%)	
	Married (n=196)			Unmarried (n=181)		
Low Scores (f %)	87(44%)	28(14%)	45.27***	47(26%)	39(22%)	3.89*
High Scores (f %)	22(11%)	59(31%)		38(21%)	57(31%)	
	Employed (n=198)			Non-employed (n=179)		
Low Scores (f %)	107(54%)	23(11%)	32.65***	27(15%)	44(25%)	3.70*
High Scores (f %)	29(15%)	39(20%)		31(17%)	77(43%)	

* p<.05,

*** p<.001

## DISCUSSION

In this study on prevalence and comorbidity of SOCD and stress in normative sample of educated adults, 377 participants participated and reported differences across gender, marital and work statuses.

### SOCD, Gender, Marital Status, and Employment Status

The past studies demonstrated mix results and reported marginal differences regarding the prevalence of SOCD in men and women.^4^,^5^ Although, no significant gender differences in SOCD symptoms were observed in the current study but few interesting findings are noticed that has not been previously examined in the local context.^12^ A significant high prevalence of contamination and washing, and obsessional thoughts of harm was exhibited by women. Contrary to that, men manifested significant high prevalence of obsessional impulses. One reason of these differences could be difference in social roles and responsibilities that men and women play in Pakistani culture.

In comparison of marital status, the current study explored high prevalence of SOCD in unmarried participants which is consistent with the literature stating that single participants are more likely to be affected than their married counterparts.^17^ This might relate with early onset of SOCD which has been found to be 21.7 years world wide^17^ and majority of study participants in present research were in their twenties 65% (21-29 years). In the context of employment status, a significant high prevalence of SOCD was observed in non-employed participants. Although no past findings comparing employed and non-employed adults are available to support/contradict this findings but a recent study concluded that students are most vulnerable group to be identified with SOCD symptoms and this could be due to the vulnerability of early onset of these symptoms.^18^

### Stress, Gender, Marital Status, and Employment Status

While exploring the gender difference regarding prevalence of stress, analysis illustrated insignificant findings. This result is contrary to some of the past studies which claimed that women are more likely to manifest high level of stress than their counterparts.^6^,^19^ This could be attributed to the difference in perception of stress as in eastern culture; it’s taken as absence of inner peace whereas, in the western countries, described as loss of control. The results in our research also demonstrated that non-employed adults reveal high level of stress than their employed counterparts. These results supplemented the past findings revealing that employed individuals experience low level of stress.^9^,^20^ This could be due to the fact that work or job related tasks facilitates individuals to improve their coping strategies to deal with stress. In addition, insignificance prevalence was noticed while comparing stress across marital status. It contradicted past observation claiming that single individuals are likely to report high level of stress due to the high cortisol level.^8^

### Comorbidity of SOCD Symptoms and Stress

SOCD and stress share several commonalities in symptomology e.g., repeated disturbing thoughts, avoidant behavior, avoiding distress causing objects, places, clues and repetitive actions to avoid anxiety.^21^ These commonalities let the researchers to establish the notion of comorbidity of SOCD and stress. This research results are in line with past inferences claiming the strong comorbidity between SOCD and stress.^12^

### Strengths and Limitations of Study

The strength of current research includes a reasonably large sample size with high response rate. This research was conducted in two top ranking universities of cosmopolitan city Lahore which widened the generalizability of findings. This strength also signifies the importance of examining SOCD symptoms in the present study as a past research on exploring prevalence of clinical nature of OCD in normative sample recruited culturally specific population.^12^

### Strength and Limitations of the Study

However, this could imply as limitation of this study as well because it included participants only from academic institutions. Although, it was on a comparatively larger sample size, yet participants from highest ranking universities may not be representative of all universities and age groups. Further studies using a larger and diversify sample from different setting are suggested to be conducted. Another, strength of current study is that sample comprised of well qualified participants who were better able to identify and rationalize obsessions, compulsions and stressful thoughts and feelings. In this study, reported high prevalence of SOCD symptoms and stress could not be taken as an indicator of clinical diagnosis as the administered tools were not diagnostic and structured only for normative samples. Another limitation of the research was that only psychological comorbidity was controlled as extraneous factor, though ignoring that comorbidity could be of psychiatric or physical nature.

## CONCLUSION

The findings of this study illustrated that SOCD symptoms and stress were widely prevalent and it varies across genders, marital and employment particularly non-employed sample manifested high prevalence of stress and SOCD. There was significant comorbidity between SOCD symptoms and stress and high rates were observed in samples of men and non-employed participants. As OCD symptoms remain unidentified, identifying young adults at high risk for developing OCD may be an important goal. Since early recognition of the problem and interventions may benefit treatment outcomes.

### Author’s Contribution

**FA** contributed in conception and designing the study, data collection, data analysis and interpretation and manuscript writing.

**SM** contributed to the statistical analysis, interpretation and final drafting the article.

**AA** helped in data collection.
